# Affordable novel device (VY) for transperineal prostate biopsy: A trial on prostate mannequin

**DOI:** 10.1016/j.mex.2021.101417

**Published:** 2021-06-18

**Authors:** Sawkar Vijay Pramod, Ferry Safriadi, Yasser Kuddah, Richardo Raditya Handoko

**Affiliations:** Urology Department, Hasan Sadikin Academic Medical Center, Faculty of Medicine, Universitas Padjajaran Bandung, Jl. Pasteur No. 38 Bandung Jawa Barat, 40161 Indonesia

**Keywords:** Prostate biopsy, Transperineal biopsy, VY

## Abstract

Prostate cancer (PCa) is the most frequent malignancy in men worldwide after lung cancer. The gold standard for diagnosing prostate cancer is prostate biopsy. There are two main approaches for diagnosing PCa: transrectal biopsy and transperineal biopsy. In Indonesia, transperineal approach is rarely done as a part of diagnostic work up.

The most known method of transperineal biopsy is the fan technique, which is a difficult technique to perform, particularly when directing the needle to the target accurately. For this reason, we develop an affordable bioplastic VY probe mounted needle guide device ($10) to provide precise needle insertion during the biopsy. In this study, we assessed the objective and subjective factors of the newly developed VY device.

• Basic knowledge and operation technique were equalized by holding a prostate biopsy training using fan technique and VY device to 20 first-year Residents
• Participants were randomized and devided into two groups to perform fan technique and VY technique based on the training given
• Subjective factors (usefulness, the easiness, and preference) and objective factors (duration of puncture to needle visualization on USG, biopsy duration time, and the ability to reach the target tissue) were evaluated.

## Graphical abstract

**Figure:**
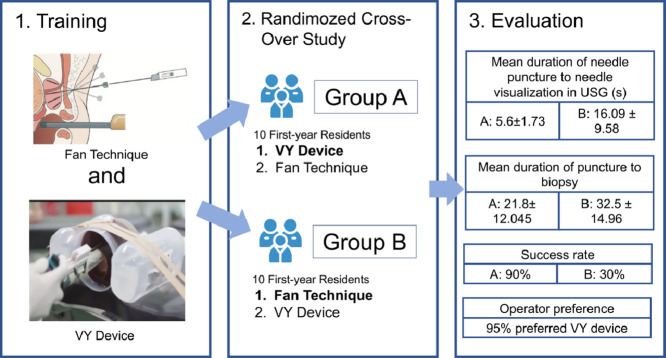

Specifications Table

**Table:** 

Subject Area:	Medicine and dentistry
More specific subject area:	A novel device for prostate biopsy
Method name:	VY Device
Name and reference of original method:	He BM, Chen R, Shi ZK, Xiao GA, Li HS, Lin HZ, et al. 2019. Trans-Perineal Template-Guided Mapping Biopsy vs. Freehand Trans-Perineal Biopsy in Chinese Patients With PSA < 20 ng/ml: Similar Cancer Detection Rate but Different Lesion Detection Rate. *Front Oncol*;9:758.
Resource availability:	https://www.frontiersin.org/articles/10.3389/fonc.2019.00758/full

## Introduction

Prostate cancer (PCa) is the most frequent malignancy in men worldwide after lung cancer, counting for 1,276,106 new cases and causing 358,989 deaths (3.8% of all deaths caused by cancer in men) in 2018 [1,2]. Incidence of prostate cancer were the highest in African – American men compared to White men [1,3]. In Indonesia, the incidence of prostate cancer was 4.5 – 9.8 per 100,000 population in 2002 and increased to 7.5 –14 per 100,000 in 2008; became the fifth most frequent malignancy in men with 11,361 (7.1%) new cases and 5,007 deaths reported in 2018 [1,^2^,^4^,5]. Previous study conducted by Safriadi et al at Hasan Sadikin Hospital Bandung, Indonesia showed an increased trend of prostate cancer cases in 2004-2011 [5].

Cancer detection is important for the management of malignancy in making a medical decision and reducing mortality. Although serum PSA level, digital rectal examination (DRE), transrectal ultrasound (TRUS), and magnetic resonance imaging (MRI) help a lot for PCa detection, a prostate biopsy is still the gold standard for confirming the histological diagnosis for now [6], [7], [8].

Ultrasound-guided biopsy remains the standard of care [8]. There are two main approaches for diagnosing PCa: transrectal biopsy and transperineal biopsy [9]. EAU Guidelines 2020 concludes that transrectal and transperineal approaches are recommended in the same manner. In Indonesia, transrectal biopsy is still the most common method. The latest Indonesian urologic guideline still proposes the transrectal biopsy approach as a recommended diagnostic tool for diagnosing PCa [10,11].

Being generally considered as a relatively low-risk outpatient approach, up to 50% of the patients suffer from minor complications (e.g. hematuria, hematospermia, rectal bleeding, and acute urine retention) to severe complications (e.g. anemia and syncope) [9]. Clinical complications and hospital admissions following TRUS prostate biopsy have increased during the last 10 years, primarily due to an increasing rate of infections [12]. Approximately 4 to 5% of patients who undergo this procedure require hospital admission due to infection-related complications (ranging from bacteriuria to sepsis) [9]. Other study concluded 1-2% rate of bacteremia, 5-8% bacteriuria, 0-8% sepsis, and 1% hospitalization. Therefore, prostate biopsy performed through other approaches should be considered [10].

The transperineal approach was introduced to improve the safety of prostate biopsy [8,13]. Standard transperineal and transrectal prostate biopsies provide a superimposable detection rate of PCa, but the transperineal approach permits the operator for easier and better reach of the anterior zone of the prostate [14]. This procedure has been shown to improve the cancer detection rate. Moreover, the transperineal approach also has a lower risk for complications [10].

Transperineal targeting of biopsy cores using fan technique without needle guide device is often limited by technical factors. Directing the biopsy needle to get the target region of the prostate is difficult [15]. A trans-perineal template-guided mapping biopsy (TTMB) is said to be easier to drive the needle for the sampling of the prostate, with a brachytherapy stepping unit and grid. The cancer detection rate in TTMB is also higher than the fan technique transperineal biopsy. However, the device makes the procedure takes more time and the equipment is more expensive [7]. In this study, the authors aim to discover a new approach using a better, more efficient, and more reasonably priced device to provide precision during the biopsy procedure. We developed a VY probe mounted needle guide device. It was made by a 3D printing machine with cornstarch-based bioplastic material commonly called poly acetic acid material, a biodegradable plastic. It had an affordable price for developing countries at about 10 USD.

The assessment of newly developed medical devices is challenging. The basic premise of introducing a new medical device is better performance than available alternatives or filling a gap where there is none. Both objective and subjective measures can be used for evaluation. Objective assessment is a relatively straightforward indicator where one device is compared with another using a measurable indicator of performance. The biopsy accuracy and operation time are the chosen indicators in evaluating VY. Subjective assessment is often the basis for the introduction of new medical technology. Subjective measures of performance include human elements (such as perceived usability and preference) that can be harder to define. While the degree to which these subjective measures influence decisions regarding technology/device implementation is complex and certainly context-specific. There is little doubt that they are important in determining the uptake and utility of medical devices in clinical practice. Furthermore, promoting subjective benefits is not a new concept with it being a core principle in the field of marketing. This study aims to assess the objective and subjective factors of the newly developed VY [16].

## Methods

**Fig. 1:**
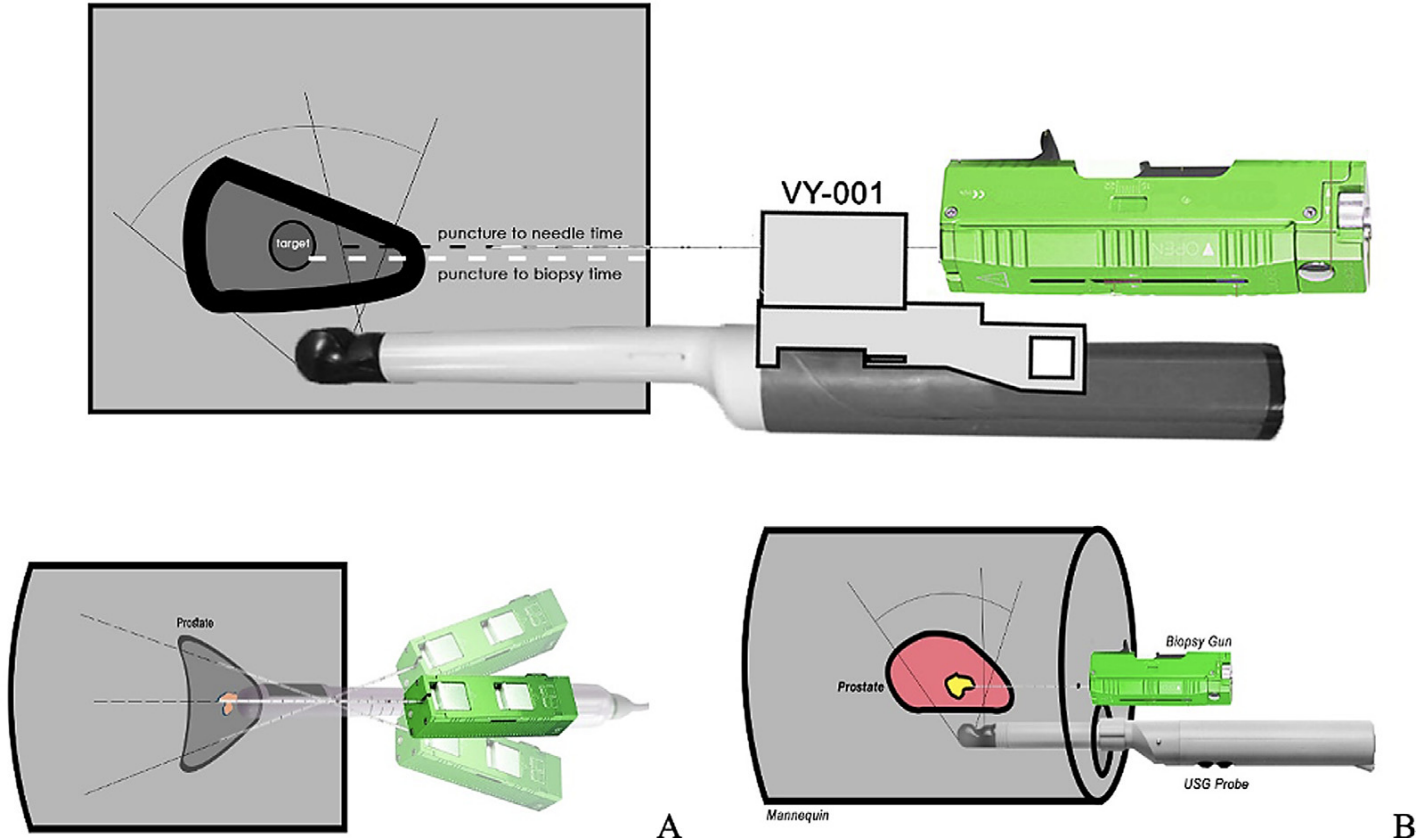
Black dotted line shows the way that needle will go parallel to the range of ultrasound probe from the skin. The white dotted line reflects the duration for needle to get the biopsy target Illustration of fan technique (no needle guide device) in prostate biopsy mannequin, anterior view (A), lateral view (B).

This is a randomized cross-over study. We do a training of VY device to the first year urology residents in Hasan Sadikin General Hospital, Bandung, so each person has the same basic knowledge and operation technique for the device. The urology residents are randomly divided and perform biopsy procedures in the prostate mannequin. The first ten residents perform a transperineal prostate biopsy using VY first then do another biopsy with fan technique (Fig. 1). The other ten residents perform a biopsy with fan technique first then do another biopsy with VY. Fan technique is performed by using a biopsy gun (PAJUNK®), biopsy needle 18G, IV catheter no 18, and transrectal ultrasonography to do a biopsy with no needle guiding tools. The results of the biopsy using the VY (Group A) and fan technique (Group B) are compared to each other.

**Fig. 2:**
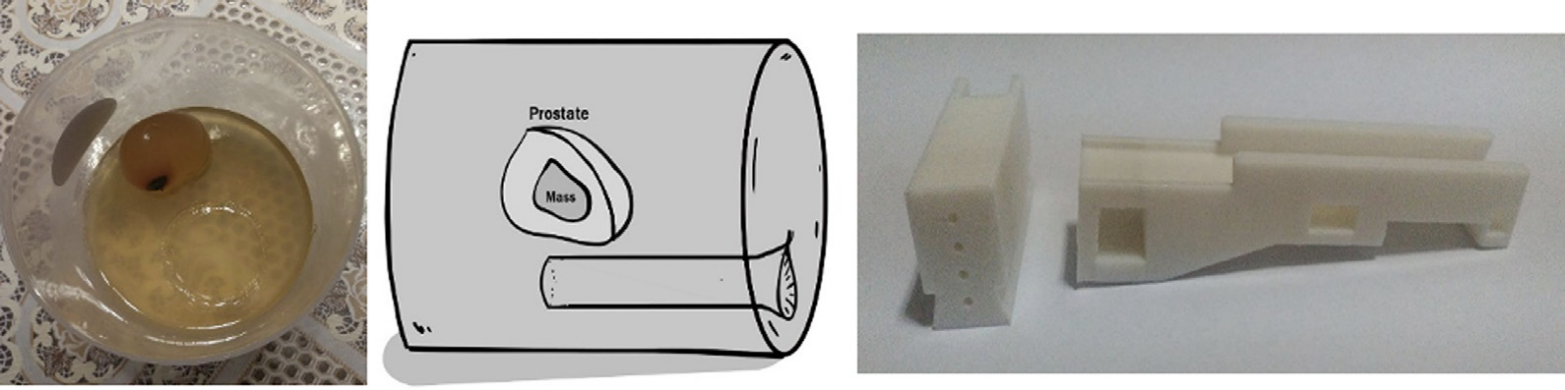
Prostate mannequin with biopsy target inside and VY device.

The mannequin is custom made from a mixture of gelatin, glycerin, sorbitol, and psyllium with a biopsy target inside (Fig. 2). The biopsy target is made from a balloon filled with a mold of gelatin and formed to be similar to the prostate anatomy. We randomly put mulberry as a targeted biopsy inside the prostate with a size of about 1-1,5 cm^3^. This study had ethical clearance from Universitas padjadjaran ethical commity wih ethical number of LB.02.01/X.6.5/55/2020. The study protocol, including methods of informed consent and protection of personal information, was approved by the Human Ethics Review Committee of Hasan Sadikin Hospital.

### Objective assessment

**Fig. 3:**
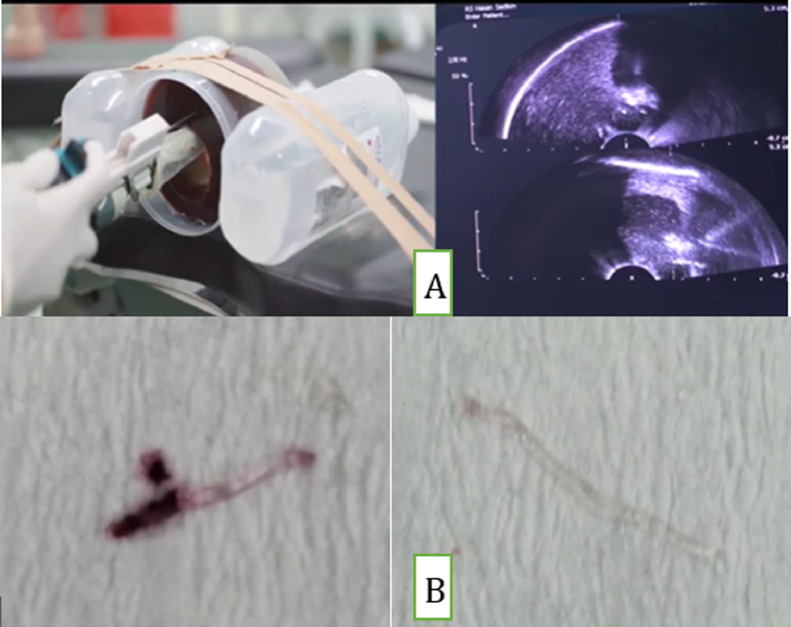
A. Biopsy with VY-001; B. Positive and negative biopsy result.

The prostate mannequin, as seen in Fig. 3, has mulberry inside as a biopsy target. The colored gelatin is added around the mulberry to make it invisible from the outside. The successful procedure is marked by the presence of the violet color of mulberry in the core biopsy specimen. The primary outcome measure consists of duration of puncture to needle visualization on USG, biopsy duration time, and the ability to reach the target tissue.

The results are compared to each other and analyzed by using Mann-Whitney. All tests are 2-sided with significance considered at p<0.05. The statistical analysis was performed using a computer program.

### Subjective assessment

Subjective perceptions from each subject were then evaluated using a questionnaire through Google Form application after completing the procedure using the two techniques. The questionnaire was adapted from the Universal Design Performance Measure for Products [17]. The modified questionnaire consists of 13 statements divided into 3 sections, perceived usefulness (4 statements), perceived ease of use (6 statements), and preference (3 statements). The subjects have to rate each statement using a linear scale, score 1 if the subjects strongly disagree with the statement and score 5 if the subjects strongly agree with the statement.

## Result

### Objective assessment

Twenty first-year urology residents in Hasan Sadikin General Hospital have participated in this study. All residents had never performed transperineal prostate biopsy before.

The mean duration of puncture to needle visualization in USG for group A and group B was 5.6±1.73 seconds and 16.09 ± 9.58 seconds respectively (p=0.000). The puncture time to needle visualization faster in 70% of residents with VY. The mean duration of puncture to biopsy (biopsy time) was 21.8± 12.045 seconds in group A and 32.5 ± 14.96 seconds for group B (p = 0.026). The biopsy duration was faster in 75% of residents with VY. Statistical analyses for both duration were done using the Mann-Whitney test. Both duration, puncture to the needle, and puncture to biopsy times are significantly faster with VY compared to biopsy with fan technique.

Fig. 3 shows that the positive biopsy revealed a violet color. With the presence of the violet color, the biopsy was called successful.

The success rate for biopsy in group A was 90 % and 30% in group B (p=0.000). Statistical analysis was also done using the SPSS program and it was significantly more accurate compared to biopsy without the device.

### Subjective assessment

**Fig. 4:**
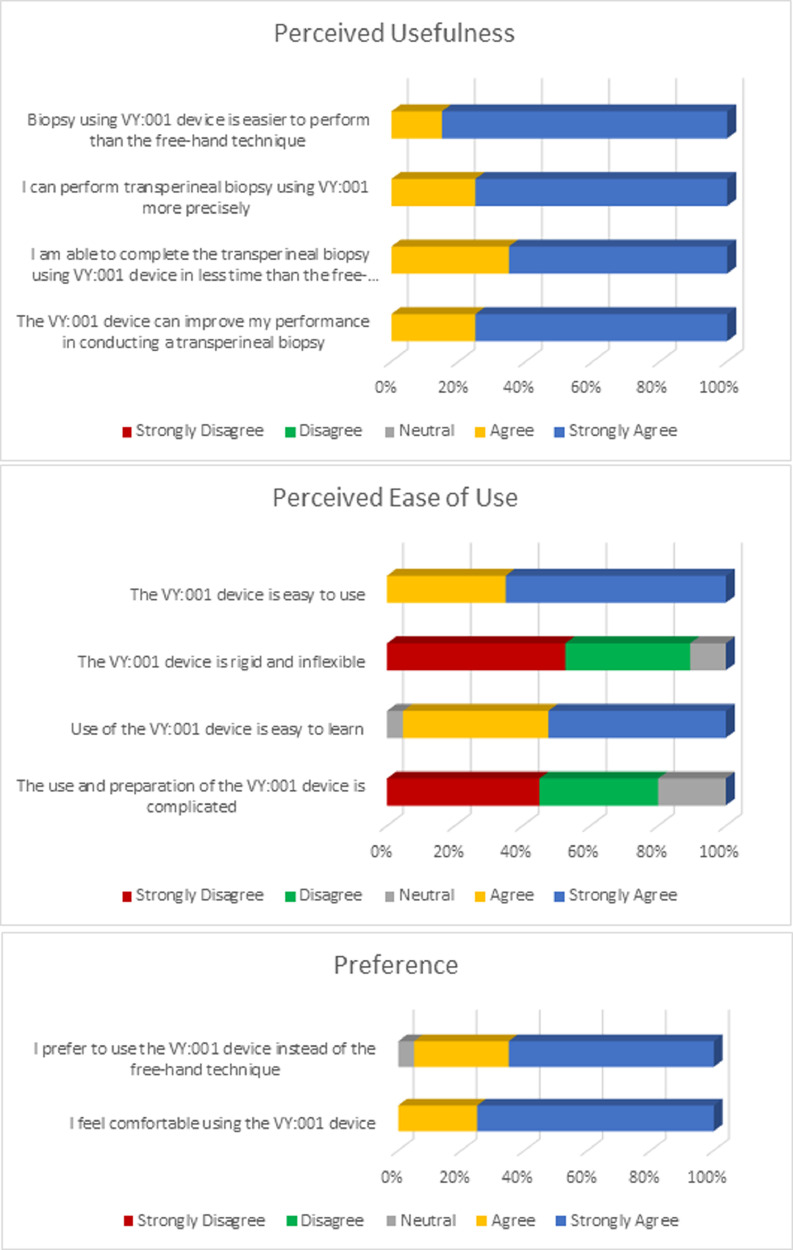
Subjective assessment.

The result of the questionnaire was divided into 3 sections of perceived usefulness, perceived ease of use, and preference as seen in Fig. 4.

### Perceived usefulness

The perceived usefulness category consisted of 4 statements. All respondents (100%) agreed that biopsy using VY device was easier to perform than the fan technique (85% strongly agree, 15% agree), performed more precisely (75% strongly agree, 25% agree), and was able to be completed in less time than the fan technique (65% strongly agree, 35% agree). All respondents (100%) agreed that the VY device could improve their performance in conducting a transperineal biopsy compared to the fan technique.

### Perceived ease of use

The result of perceived ease of use section showed that the device was easy to use (65% strongly agree, 35% agree), had good flexibility (50% strongly agree, 35% agree), and was easy to be learned by first time user (55% strongly agree, 40% agree). Most respondents (80%) thought that the preparation of VY device was not complicated (45% strongly agree, 35% agree).

### Preference

Most respondents (95%) preferred to use the VY device instead of the fan technique in performing a transperineal biopsy (65% strongly agree, 30% agree). All respondents (100%) felt comfortable performing transperineal prostate biopsy using the VY device (75% strongly agree, 25% agree).

## Discussion

In this study, the device VY can help urology residents to perform transperineal prostate biopsy faster and more accurate. Both duration of the needle to be visualized in USG screen and duration of biopsy are significantly faster with VY device. The mean duration for the needle to be visualized in USG in this study is almost one-third of the duration without the device. In the fan technique, the needle usually did not go in line with the ultrasonography picture range, so it takes skill, and not everyone could do it well. But with VY, the position of the needle easily visualize even for the first-year student with no experience of prostate biopsy [1]. Some studies conclude that the detection rate for prostate cancer with the transperineal approach of an experienced urologist compared with resident results were 64.5% and 30% respectively [18]. But with VY, we believe in cutting the learning curve for better result of prostate biopsy.

This advantage is also supported by the accuracy of the device to take a biopsy sample from the tissue [19]. In this case, the device can significantly improve the positive rate of biopsy. The VY device ensures precise targeting of almost all areas of the prostate as the biopsy target was put in a random way inside the prostate; while other study said that biopsy with fan technique lacked biopsy cores in some parts of the prostate, like the central zone under the urethra, the left and right anterior section and the section from mid-gland to the base [2].

Other studies reported that by using the PrecisionPoint device, the anterior prostate was successfully sampled in all cases. In total, cancer was detected in the anterior peripheral zone in 8 (18.6%) cases, with cancer detected exclusively in this region in 2 (4.7%) men. These data were notable, as it had long been appreciated that the anterior prostate was difficult to access with the transrectal approach and tumors arising from this location had a propensity for a higher grade and stage [20].

This study confirmed that VY could significantly improve the duration for transperineal prostate biopsy and also had better accuracy compared to biopsy without the device. This VY was made from 3D printing which is cheaper and more easily accessible for all-region. Other USG mounted devices are expensive and not available in Indonesia. A further experimental study to compare VY with other USG mounted device is needed.

The assessment of newly developed medical devices is challenging. The basic premise of introducing a new medical device is better performance than available alternatives or filling a gap where there is none. Subjective measures of performance include human elements (perceived usability and preference) that can be harder to define. Subjective perceptions from each subject were then evaluated using a questionnaire.

The questionnaire was divided into 3 sections of perceived usefulness, perceived ease of use, and preference. In the perceived usefulness section, the respondents assessed the device's performance during the procedure. All respondents (100%) agreed that transperineal prostate biopsy using the VY device was easier and could be precisely performed than the fan technique. All respondents (100%) agreed that the VY device could improve their performance in conducting a transperineal biopsy in less operating time.

The perceived ease of use section assessed the characteristics of the VY device. Most respondents agreed that the device was easy to use, had good flexibility, and was easy to be learned by the first-time user. The preparation of the VY device was also not complicated.

The preference section assessed the user perspective while performing a transperineal prostate biopsy using the VY device. Most respondents preferred to use the VY device and felt comfortable performing transperineal prostate biopsy using the VY device instead of the fan technique.

## Conclusion

In our experience, transperineal prostate biopsy with VY is better than the fan technique compared to biopsy duration and accuracy at the prostate model. The device is versatile and easy to use. Most subjects preferred to perform a transperineal prostate biopsy using the VY device instead of the fan technique. In the future, further clinical practice is needed to confirm the result.

## Declaration of Competing Interest

This study had no conflict of interest.
